# Primary Pancreatic Large B-Cell Lymphoma Presenting as Acute Pancreatitis

**DOI:** 10.7759/cureus.9583

**Published:** 2020-08-06

**Authors:** Alay Tikue, Genanew Bedanie, Luis Brandi, Sameer Islam, Kenneth Nugent

**Affiliations:** 1 Internal Medicine, Texas Tech University Health Sciences Center, Lubbock, USA; 2 Pathology, Texas Tech University Health Sciences Center, Lubbock, USA; 3 Gastroenterology and Hepatology, University Medical Center, Lubbock, USA; 4 Internal Medicine/Pulmonary and Critical Care Medicine, Texas Tech University Health Sciences Center, Lubbock, USA

**Keywords:** non-hodgkin lymphoma, primary pancreatic lymphoma, diffuse large b cell lymphoma, acute pancreatitis

## Abstract

Primary pancreatic lymphoma (PPL) is an extremely rare form of extranodal malignant lymphoma. The most common histological subtype of PPL is diffuse large B-cell lymphoma (DLBCL). Clinical and imaging features of PPL may often overlap with pancreatic adenocarcinoma. Therefore, it is very important to obtain a preoperative cytohistology diagnosis of pancreatic tumors to avoid unnecessary surgeries in cases with a diagnosis of PPL.

Herein, we report a 71-year-old male who was admitted to our hospital with a diagnosis of acute pancreatitis after he presented with complaints of nausea, vomiting, and epigastric abdominal pain. MRI of the abdomen revealed a pancreatic head mass, and histopathology and immunohistochemical assessment of the pancreatic lesion established the diagnosis of DLBCL. The patient achieved remission after six cycles of rituximab-cyclophosphamide, doxorubicin (hydroxydaunomycin), vincristine (oncovin), prednisolone (R-CHOP) chemotherapy.

## Introduction

Pancreatic malignancies are among the common cancers and account for one-fourth of cancer-related deaths in the United States [1]. The majority of pancreatic malignancies includes adenocarcinomas; hence, clinicians are not well aware of other subtypes of pancreatic neoplasms [2-3]. Primary pancreatic lymphoma (PPL) is a rare form of extranodal malignant lymphoma which accounts for less than 1% of all primary pancreatic tumors. Even though gastrointestinal lymphomas encompass to third of all extranodal lymphomas, the involvement of the pancreases is extremely rare [4].. The most common histological subtype of PPL is diffuse large B-cell lymphoma (DLBCL) [5].

Presenting symptoms and imaging modalities are nonspecific and may lead to delays in diagnosis. The common presenting symptoms include abdominal pain, bowel obstruction, obstructive jaundice, weight loss, and rarely acute pancreatitis [6-7]. Although PPL is a very rare cancer of the pancreas, making the diagnosis has paramount importance due to its amenability to treatment even in very advanced stages. Therefore, histological diagnosis is mandatory because of the different prognosis and therapeutic approaches of these different types of pancreatic tumors [8].

In this article, we report a case of PPL in an elderly man who presented with symptoms of acute pancreatitis.

## Case presentation

A 71-year-old white man with diabetes mellitus and hypertension presented to the hospital with nausea, vomiting, and abdominal pain which was located in the epigastrium, constant, sharp in quality, 8/10 in intensity, and radiating to the back for the last three days. He had a loss of appetite and weight loss for two months. He denied diarrhea, constipation, melena, hematemesis, jaundice, or abdominal distension.

**Figure 1:**
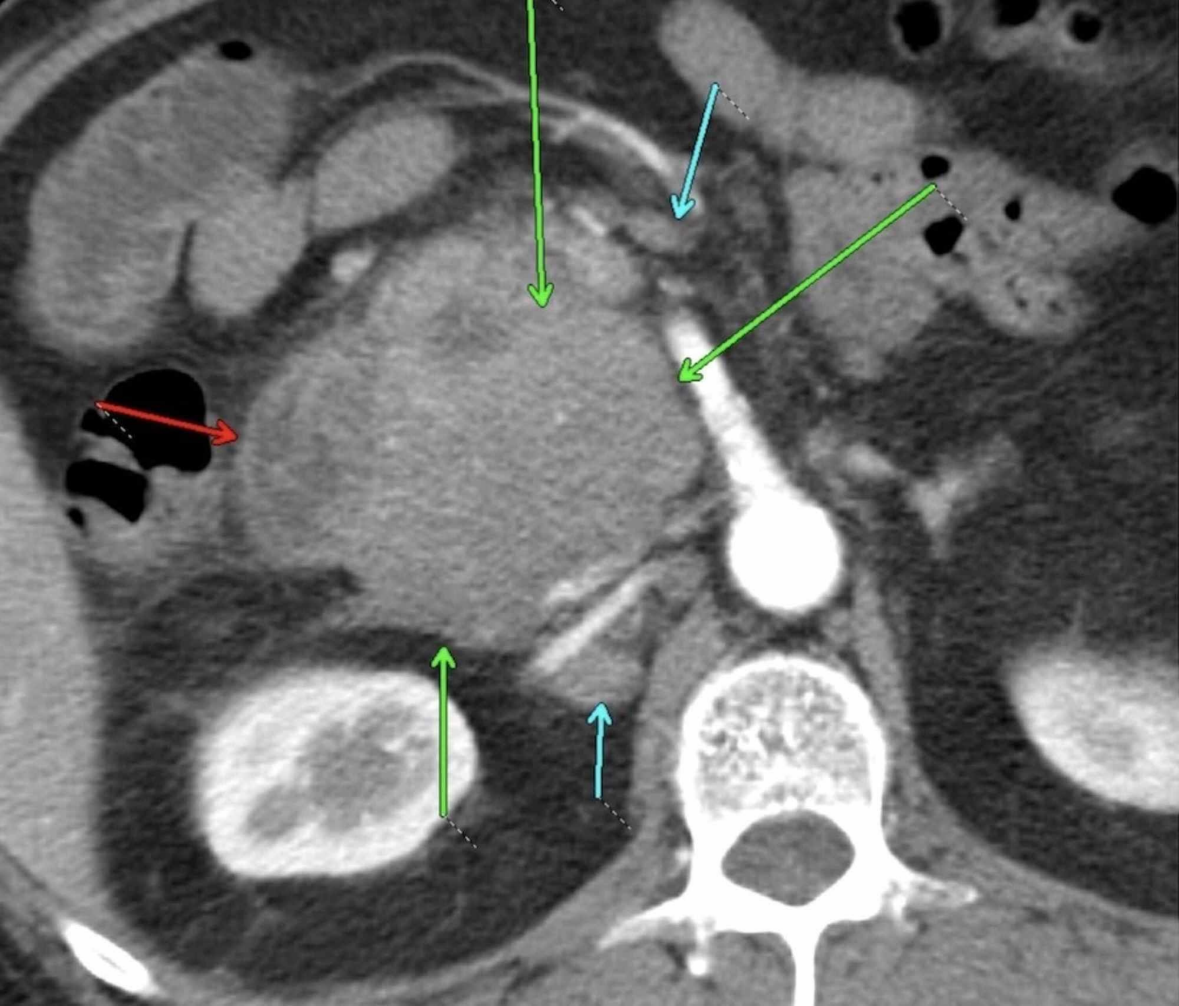
Contrast enhanced axial image through the abdomen shows homogenous confluent mass at the pancreatic head (green arrows), with the duodenum (red arrow), and the surrounding lymphadenopathy (cyan arrows).

**Figure 2:**
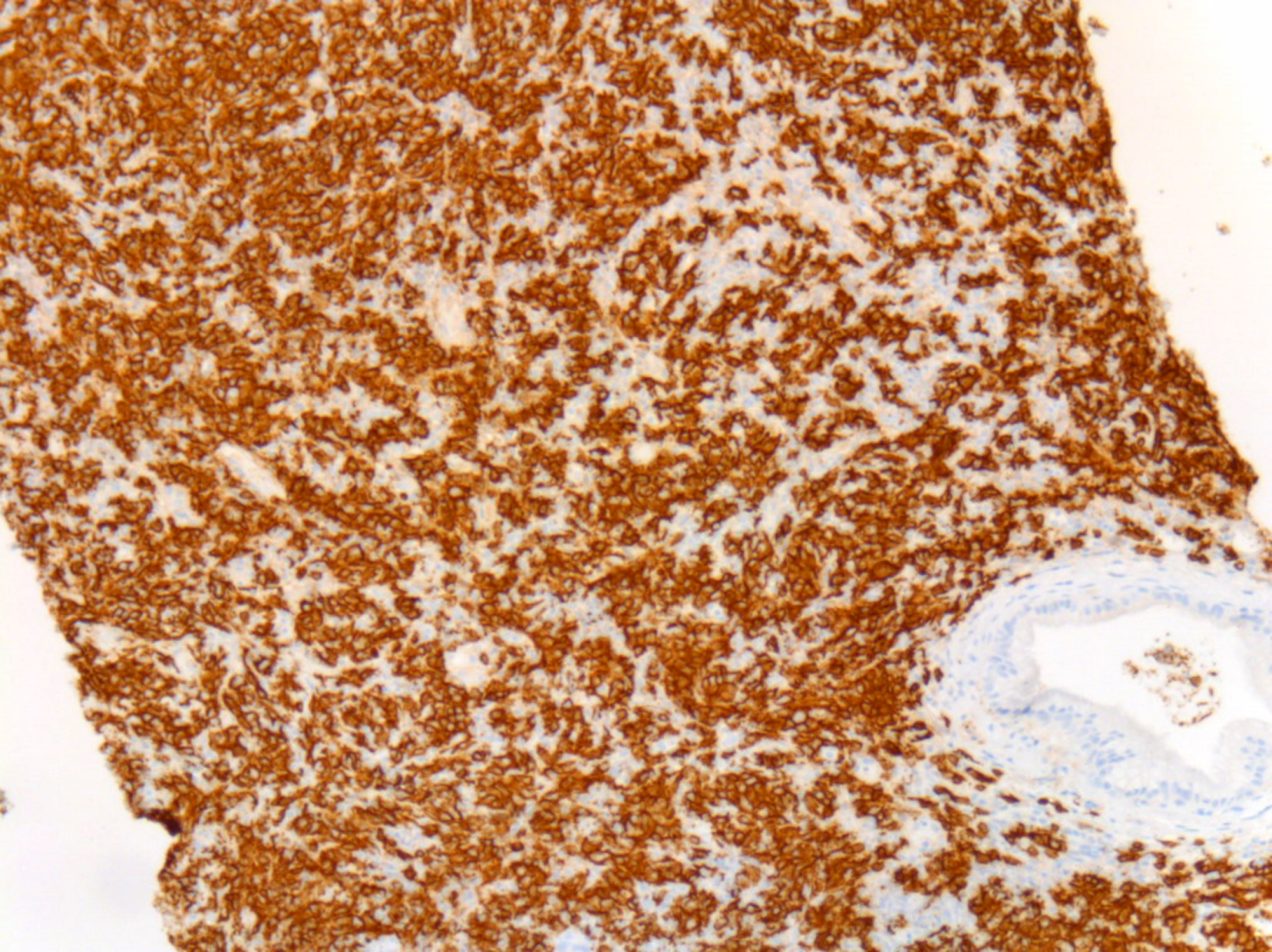
Low power magnification of CD20 antibody staining shows a diffuse B-cell population, an abnormal finding.

**Figure 3:**
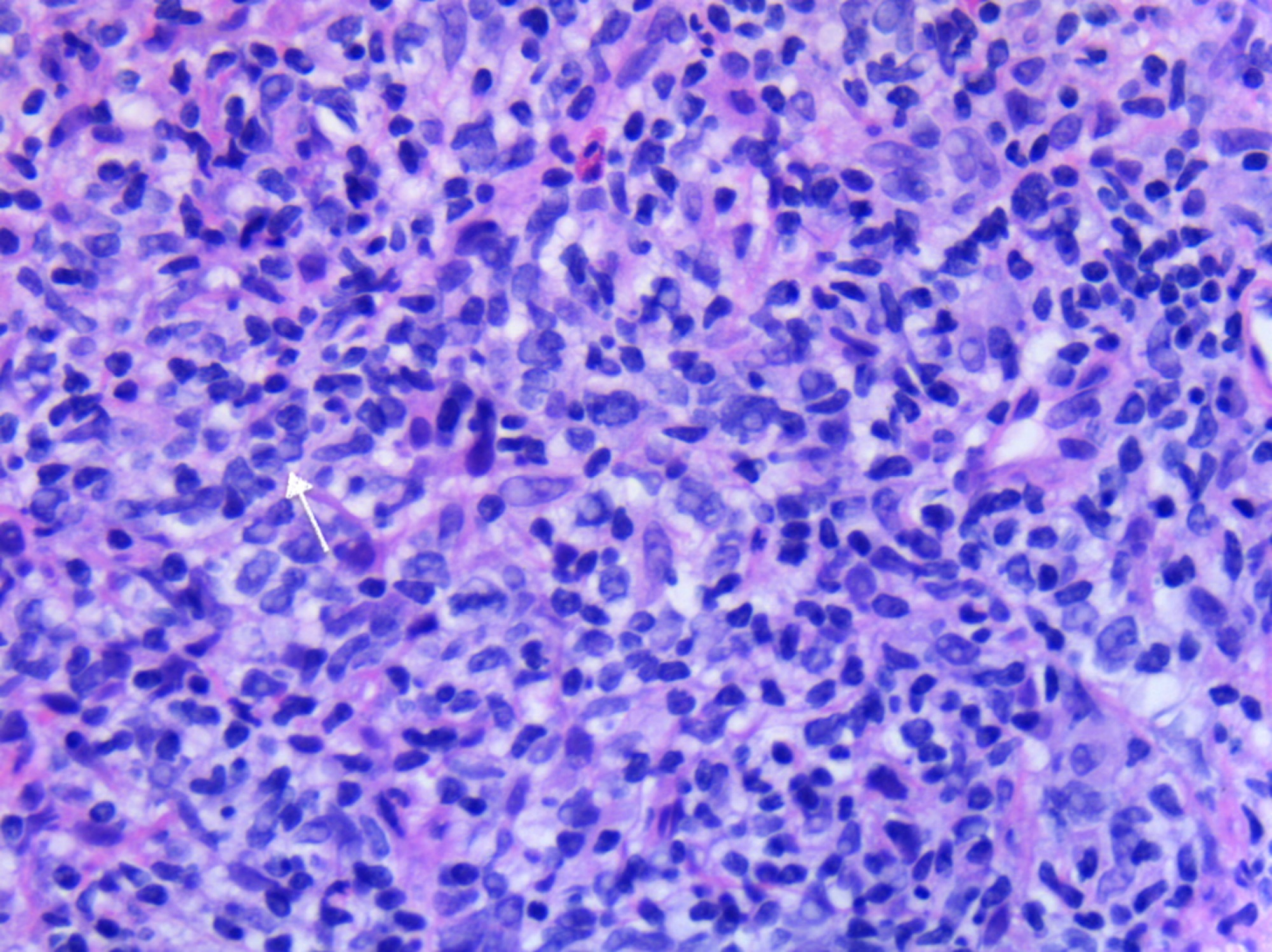
White arrow points to mitotic figure and immediately at 10:00 adjacent can be seen a neoplastic B-cell.

On physical examination, his vital signs were normal, and he had tenderness on palpation at the epigastric area without signs of peritonitis. Complete blood counts were within normal limits. He had transaminitis and elevated total bilirubin and alkaline phosphatase. Serum amylase and lipase were 123 IU/L (normal: 13-53) and 864 IU/L (normal: 13-60), respectively. Tumor markers, including carbohydrate antigen (CA 19.9), carcinoembryonic antigen (CEA), and alpha-fetoprotein (AFP) were normal. MRI of the pancreas showed a large mass at the pancreatic head measuring 8.3 cm x 7.6 cm in maximum diameter with peripancreatic and retroperitoneal adenopathy (Figure 1). Endoscopic ultrasound demonstrated the mass in the pancreatic head and malignant-appearing peripancreatic lymph nodes. CT guided biopsy of the pancreatic mass was performed. Pathology revealed a high-grade B-cell lymphoma (Figures 2-3). Bone marrow biopsy did not show atypical cells. Staging CT scans revealed a 9 mm sized mediastinal node.

He was diagnosed with stage III high-grade B-cell PPL with an international prognostic index of two which predicts a five-year survival of 51%. He received a rituximab-cyclophosphamide, doxorubicin (hydroxydaunomycin), vincristine (oncovin), prednisolone (R-CHOP) regimen for treatment and is currently on cycle six with a good response. During the total six cycles of chemotherapy, he did not have any major treatment-related complications except for transient transaminitis during the second cycle which resolved without the need for chemotherapy dose modification. 

Currently, he follows with oncology at the outpatient cancer center and overall, he is doing well. A surveillance CT of the abdomen, pelvis, and chest at six months and one year postchemotherapy did not show any evidence for disease recurrence. Chemistry work-up at one-year postchemotherapy showed C-peptide and hemoglobin A1C in the normal limits. 

## Discussion

Pancreatic adenocarcinoma is the most common pancreatic tumor which accounts for 85% of these tumors; a very small proportion is due to PPL. This is a rare malignancy accounting for 0.7% of all pancreatic malignancies and 1% of extranodal lymphomas [9- 10]. In a review by Saif in 2006, fewer than 150 cases of PPL have been reported in English literature at that time and the majority of our information about PPL is from single case reports [6-7]. The disease is seven times more common in male and the average age at presentation is 57 [11-12]. Nonspecific gastrointestinal symptoms are the most common presentation; these include abdominal pain, obstructive jaundice, diarrhea, weight loss, and acute pancreatitis [11]. For unclear reasons, the classic B symptoms of nodal non-Hodgkin lymphoma are very rare in patients with PPL [12]. Our case presented with sign and symptoms of acute pancreatitis.

It is imperative to differentiate PPL from adenocarcinoma when a pancreatic mass is diagnosed, as the type of mass affects both treatment options as well as outcomes [12]. Some imaging characteristics of the pancreatic mass may help to distinguish PPL from the more common pancreatic adenocarcinoma [13]. But radiological studies are not a definitive method in distinguishing PPL from pancreatic adenocarcinoma [10, 14-15]. Therefore, preoperative cytohistology of the pancreatic mass is required to diagnose, to guide definitive treatment, and to avoid unnecessary surgery. An accurate CT guided fine-needle aspiration (FNA) diagnosis of PPL is critical for timely, nonsurgical management. Flow cytometry and immunohistochemistry have significantly enhanced the diagnostic role of FNA [7, 10-11].

Chemotherapy alone is the main treatment of PPL with very good long-term remission. Surgery has a small role in management PPL and is limited to cases in which, FNA along with flow cytometry is nondiagnostic and the nature of the pancreatic mass remains uncertain [16-17]. The most common chemotherapy regimen includes R-CHOP [18]. PPL cases managed with comprehensive treatment approaches have a 30% cure rate compared to a 5% five-year survival rate of pancreatic adenocarcinoma [7, 16].

## Conclusions

Primary pancreatic lymphoma is a rare form of extranodal malignant lymphoma and the patient can present with nonspecific symptoms which may lead to delays in diagnosis. Our patient presented with symptoms of acute pancreatitis but imaging as a part of acute pancreatitis work up revealed pancreatic mass. Even though mortality related to pancreatic cancer is very high, it is also very important to consider other possible differential diagnoses like PPL which has a better outcome with treatment. Therefore, we want to emphasize the importance of obtaining preoperative cytohistology of the pancreatic mass to diagnose, to guide definitive treatment, and to avoid unnecessary surgery.
